# Clinical significance of prognostic nutritional index in patients with glioblastomas

**DOI:** 10.1097/MD.0000000000013218

**Published:** 2018-11-30

**Authors:** Jin-Duo Ding, Kun Yao, Peng-Fei Wang, Chang-Xiang Yan

**Affiliations:** a Department of Neurosurgery; b Department of Pathology, Sanbo Brain Hospital, Capital Medical University, China.

**Keywords:** glioblastomas, isocitric dehydrogenase mutations, prognosis, prognostic nutritional index

## Abstract

Preoperative prognostic nutritional index (PNI) has been proven to be associated with clinical outcomes in patients with malignancies. However, data regarding the role of PNI in human glioblastomas (GBMs) is lacking. We, therefore, aimed to investigate the association between PNI and clinical parameters and survival in GBM patients.

This retrospective analysis included 300 GBM patients who were surgically treated at our institute from 2008 to 2017. PNI was calculated as albumin (g/L) + 5×total lymphocyte count (10^9^/L). SPSS 22.0, GraphPad Prism 5, and X tile were the primary tools used for data analysis, figuring drawing, and calculating optimal cutoffs, respectively.

Mean albumin value, lymphocyte count, and PNI were 42.13 ± 4.43 g/L, 1.73 ± 0.71 × 10^9^/L, and 50.80 ± 6.01, respectively. PNI was increased in patients aged ≤60 years and in men. Moreover, PNI ≥44 was associated with improved overall survival in younger patients and women. PNI was not associated with isocitric dehydrogenase (IDH)-1^R132H^ mutations or predicted survival in GBM patients without such mutations. Univariate analysis showed that a high preoperative Karnofsky performance score, gross total resection, completed chemoradiotherapy, IDH-1^R132H^ mutations, and higher PNI levels were associated with favorable outcomes. Multivariate analysis showed that only completed chemoradiotherapy and IDH-1^R132H^ mutations were independent prognostic factors.

Our results indicated that PNI is associated with age and sex in GBM patients but fails to provide independent prognostic values.

## Abstract 2

Supplemental Digital Content is available in the text

## 1 Introduction

Central nervous system (CNS) tumors are relatively rare neoplasms, with an estimated 23,800 new cases and 16,700 existing cases reported in 2017.^[1]^ In previous studies, glioblastomas (GBMs) constituted the majority of CNS tumors and were associated with poor clinical outcomes.^[2,3]^ GBM patients with an isocitric dehydrogenase (IDH)-1^R132H^ mutation had longer survival rates than did those with the wild-type.^[3]^ While molecular pathology results have served as predictive factors, blood markers related to inflammation,^[4]^ coagulation,^[5]^ and albumin^[6,7]^ have also been used to predict GBM patients’ survival. Additionally, the prognostic nutritional index (PNI), calculated by combining albumin levels and lymphocyte counts, has been shown to be another predictive prognostic factor for cancers.^[8–10]^

Several studies have indicated that nutrition status is a key factor influencing GBM patients’ survival. Increased plasma albumin levels have been correlated with favorable clinical outcomes in patients with GBMs.^[6,7]^ Moreover, survival rate has been reported to be reduced in GBM patients who are either under- or overweight.^[11]^ Xu et al^[12]^ and Zhou et al^[13]^ observed that PNI was an independent prognostic marker in GBM patients. However, inadequate data have been reported regarding the association between PNI and molecular subtypes of GBMs. Therefore, retrospectively investigated the prognostic value of PNIs in GBM patients, including a consideration of IDH status.

## 2 Methods

### 2.1 Study population

This retrospective study included 300 patients who underwent surgery at Sanbo Brain Hospital, Beijing, China from 2008 to 2015. All the patients were diagnosed with GBMs according to pathology results^[3]^ and provided written informed consent. The mutation of IDH-1^R132H^ was identified by immunohistochemistry (IHC) according to our previous report.^[14]^ Adjuvant chemoradiotherapy was performed according to the Stupp protocol.^[15]^ The adjuvant therapy was considered complete when patients had received chemoradiotherapy plus an additional 2 cycles that included temozolomide.^[16]^ PNI was calculated as the total of albumin (g/L) plus 5× total lymphocyte count (10^9^/L). We excluded patients who had been diagnosed with diabetes, metabolism disease, hypertension, heart disease, autoimmune disease, or infection within the previous 3 months, as in a previous study.^[12]^ We calculated overall survival (OS) in months, and defined it as the period from operation to death or censorship. Our follow-up ended in December 2017, and no patients were lost. The ethics committee of Sanbo Brain Hospital approved all study procedures.

### 2.2 Statistical analysis

SPSS 22.0 (IBM, Armonk, NY), GraphPad Prism 5 (GraphPad Software Inc., San Diego, CA), and X title ^[17]^ software systems were used to analyze data, draw figures, and find the optimal cutoff for PNI in predicting OS, respectively. We used the Student *t* test when comparing PNI values between 2 groups. The correlation between PNI and age was analyzed with the Spearman test.^[18]^ Survival analysis was applied using the Kaplan–Meier method and log-rank test. Cox proportional hazards models were adopted for the calculation of the hazard ratios (HRs) of death in GBM patients with regard to univariable or multivariable analysis. A *P*-value of <.05 was considered statistically significant.

## 3 Results

### 3.1 Patient characteristics

We included 300 GBM patients in our study; their clinicopathological data are presented in Table 1. There were 246 primary GBMs and 54 secondary GBMs. The mean age of the patients was 49.9 ± 14.02 years, and 39.3% (118/300) of patients were women. Moreover, 66% (198/300) of patients scored ≥70 on Karnofsky performance score (KPS), and 63.4% (184/300) of patients had gross total resections (GTRs). Postoperatively, 58.3% (165/300) of patients completed chemoradiotherapy. Unfortunately, 66.3% (199/300) of patients died by the time of our last follow-up. IDH-1^R132H^ mutations were detected in 25% (75/300) of patients. Preoperatively, the mean serum albumin level and lymphocyte count were 42.13 ± 4.43 g/L (range: 25.7–52.7 g/L) and 1.73 ± 0.71 × 10^9^/L (range: 0.28–5.22 × 10^9^/L), respectively. The mean PNI level was calculated as 50.80 ± 6.01 (range: 34.2–76.2).

**Table 1:**
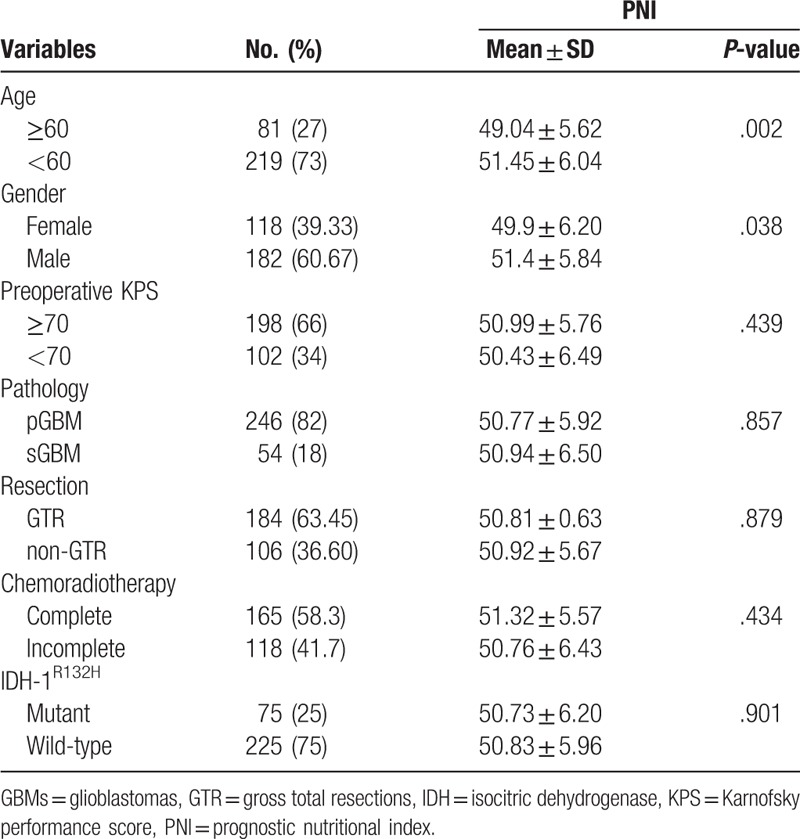
Association between PNI and clinicopathological factors.

### 3.2 Clinicopathological features and PNI

We compared preoperative PNI levels with clinicopathological factors using independent *t* tests (Table 2). Interestingly, PNI was significantly increased in patients aged <60 years (*P* = .002) and men (*P* = .038). Thus, PNI was significantly associated with age in GBM patients (*r* = −0.174, *P* = .002, Supplemental Figure 1). No differences were found in PNI levels among KPS, tumor type, or IDH-1^R132H^ status. Furthermore, PNI levels did not differ among patients who had GTR or had completed chemoradiotherapy.

**Table 2:**
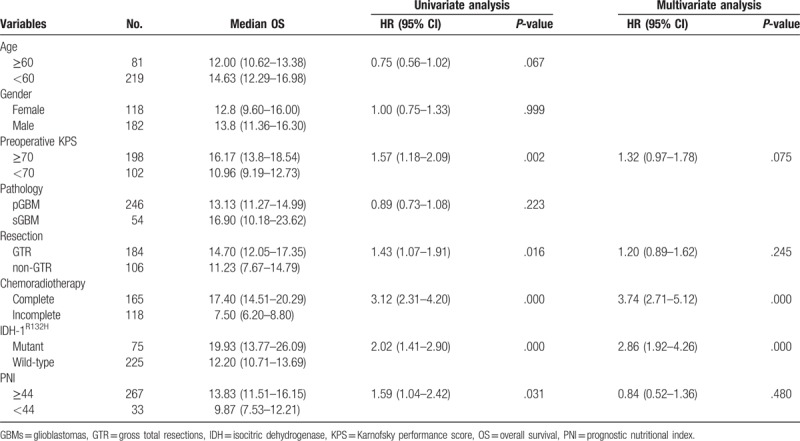
Univariate and multivariate analyses of clinicopathological factors in predicting OS in GBMs.

### 3.3 Survival analysis of PNI in GBM patients

**Figure 1:**
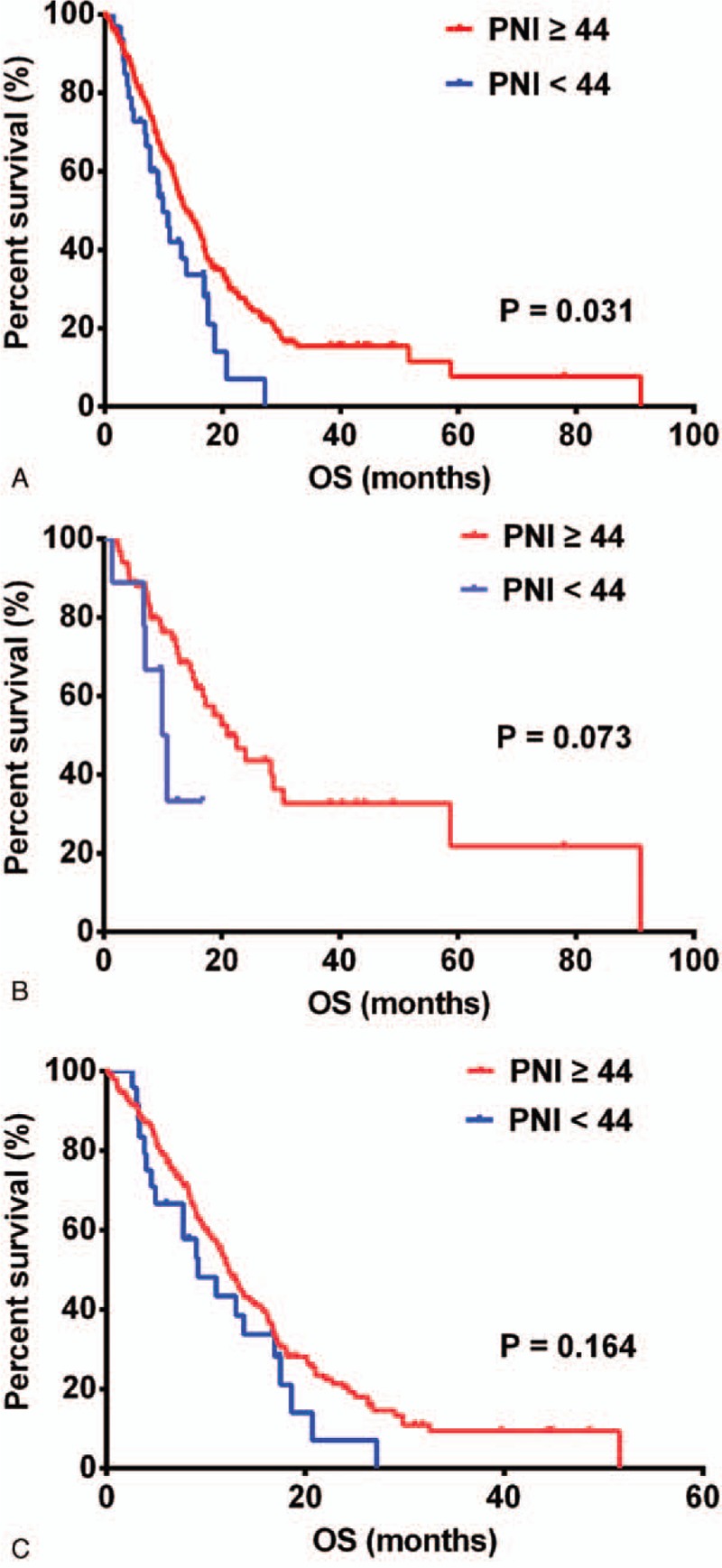
Prognostic nutritional index (PNI) predicted overall survival in (A) glioblastomas (n = 300), (B) IDH-1^R132H^ mutations (n = 225), and (C) IDH-1^R132H^ wild-type (n = 75).

The median OS (mOS) of 13.83 months (95% confidence interval [CI], 11.51–16.15) in patients with PNI ≥44 was significantly greater than the mOS of 9.87 months (95% CI, 7.53–12.21) observed in patients with PNI <44 (*P* = .031, Fig. 1A) for all GBM patients. Next, GBMs were divided into 2 IDH subtypes. In GBM patients with wild-type IDH-1^R132H^, a PNI of ≥44 was associated with a favorable outcome, although it failed to achieve significance (PNI ≥44, 20.87 months [95% CI, 14.22–27.52] vs PNI <44, 9.87 months [95% CI, 5.85–13.89]) (*P* = .073, Fig. 1B). In the group of IDH-1^R132H^ mutations, PNI did not indicate improvement in OS with statistical significance (PNI ≥44, 12.27 months [95% CI, 10.76–13.79] vs PNI <44, 9.23 months [95% CI, 4.58–13.89]) (*P* = .164, Fig. 1C).

**Figure 2:**
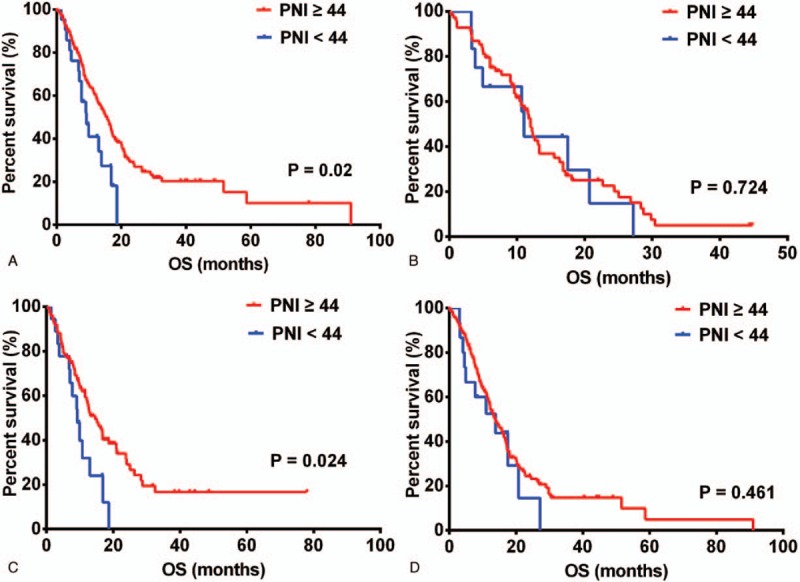
PNI predicted overall survival in (A) patients aged ≥60 years (n = 81), (B) patients <60 years old (n = 219), (C) women (n = 118), and (D) men (n = 182). PNI = prognostic nutritional index.

As preoperative PNI levels differed with age and sex, we investigated the prognostic influence of PNI based on both factors. A PNI ≥44 was associated with improved OS in patients aged <60 years (15.57 months [95% CI, 13.22–17.92]) versus those aged ≥60 years (9.23 months [95% CI, 6.33–12.13]) (*P* = .02, Fig. 2A) and in women (14.63 months [95% CI, 10.77–18.49]) versus men (9.23 months [95% CI, 6.65–11.81]) (*P* = .024, Fig. 2C). However, PNI failed to serve as a prognostic factor in patients aged ≥60 years (12.00 months [95% CI, 10.71–13.29]) versus those aged <60 years (10.96 months [95% CI, 10.16–11.76]) (*P* = .724, Fig. 2B), and in men (13.83 months [95% CI, 11.29–16.37]) versus women (13.83 months [95% CI, 3.38–23.88]) (*P* = .461, Fig. 2D).

Univariate analysis indicated that preoperative KPS, GTR, completed chemoradiotherapy, IDH-1^R132H^ mutations, and PNI levels were prognostic factors in GBM patients. However, multivariate analysis showed that only completed chemoradiotherapy and the IDH-1^R132H^ mutation were independent prognostic markers (Table 2).

## 4 Discussion

Recently, nutrition oncology has garnered great attention as playing a key role in cancer epidemiology, progression, and treatment.^[19,20]^ In this study, calculation of PNI was based on albumin levels and lymphocyte counts, which reflect both nutrition and inflammation status. PNI's prognostic value has been proven for various cancers, including colorectal cancer,^[21]^ hepatic cancer,^[22]^ and urothelial carcinoma.^[9]^ Moreover, this prognostic factor is easy to obtain, non-invasive, and could be widely used in clinics.

In this study comprising 300 GBMs cases, we first analyzed the clinical significance of PNIs, including IDH-1^R132H^ status. Our results were similar to previous reports in which a higher PNI predicted better OS in GBM patients.^[12,13]^ However, we found that poor nutrition status was strongly associated with elderly GBM patients—results inconsistent with those of previous reports.^[12,13]^ Neither was the association we found between PNI and age consistent with that among other tumors.^[21,22]^ In both our study and that of Zhou et al,^[13]^ PNI was observed to be higher in men than in women—contrary to Xu et al.'s report.^[12]^ The differing results among GBM patients were likely due to varying cut-off values and treatment strategies. Notably, we are the first to show that PNI predicts survival more accurately in GBM patients younger than 60 years and women, than in elders or men—results consistent with those of a recent report.^[23]^ Altogether, these findings suggest a strong association between PNI and age and sex.

The mechanism behind the relationship between PNI and prolonged OS in GBM patients is primarily due to albumin levels and lymphocyte counts. Albumin has been proven to be a prognostic marker in GBM patients.^[6]^ Moreover, plasma levels of albumin could reflect systemic inflammation status, because albumin expression is inhibited by TNFα and IL-6.^[24]^ TNFα and IL-6 have been shown to mediate resistance to cytotoxicity of immune cells in GBM patients.^[25]^ Lymphocyte counts and function could be negatively influenced by inflammatory cytokines.^[26]^ However, subtypes of lymphocytes, such as regulating T cells, have been shown to be immunosuppressive and associated with decreased survival rates in GBM patients.^[27]^ Therefore, we suggest that PNIs would be more accurate if lymphocyte counts excluded regulating T cells.

A higher PNI has been proven to be associated with a favorable outcome in GBM patients; however, it is unknown if survival outcomes could be improved by nutrition support in these patients. In gastric cancers, nutrition support has been shown to bring survival benefits to patients with poor nutritional status.^[28]^ Moreover, improvement in nutrition status in patients with pancreatic cancers has been associated with prolonged survivals.^[29]^ Therefore, nutrition support might potentially be of benefit to GBM patients; however, further studies were needed.

In summary, our results showed that a high PNI correlates with younger age (<60 years), female sex, and better OS. However, this conclusion needs to be confirmed in a large prospective study. Moreover, we suggest that PNI could be introduced into clinical practice as a prognostic marker, as well as a therapeutic target, to improve outcomes of GBM patients.

## Author contributions

**Conceptualization:** Changxiang Yan.

**Data curation:** Jinduo Ding, Kun Yao, Pengfei Wang.

**Formal analysis:** Jinduo Ding.

**Funding acquisition:** Changxiang Yan.

**Investigation:** Kun Yao.

**Methodology:** Jinduo Ding, Kun Yao, Pengfei Wang.

**Resources:** Changxiang Yan.

**Software:** Pengfei Wang.

**Supervision:** Changxiang Yan.

**Writing – original draft:** Jinduo Ding, Pengfei Wang.

**Writing – review & editing:** Pengfei Wang, Changxiang Yan.

## Supplementary Material

Supplemental Digital Content
