# Supplementary figures and images for: Clinical significance of prognostic nutritional index in patients with glioblastomas
Source: Medicine (Baltimore). 2018 Nov 30;97(48):e13218. doi: 10.1097/MD.0000000000013218 (PMC6283194; doi:10.1097/MD.0000000000013218)

**Supplemental Figure 1.** PNI is negatively correlated with age in GBM patients (r░=░-0.174, p░=░0.002)


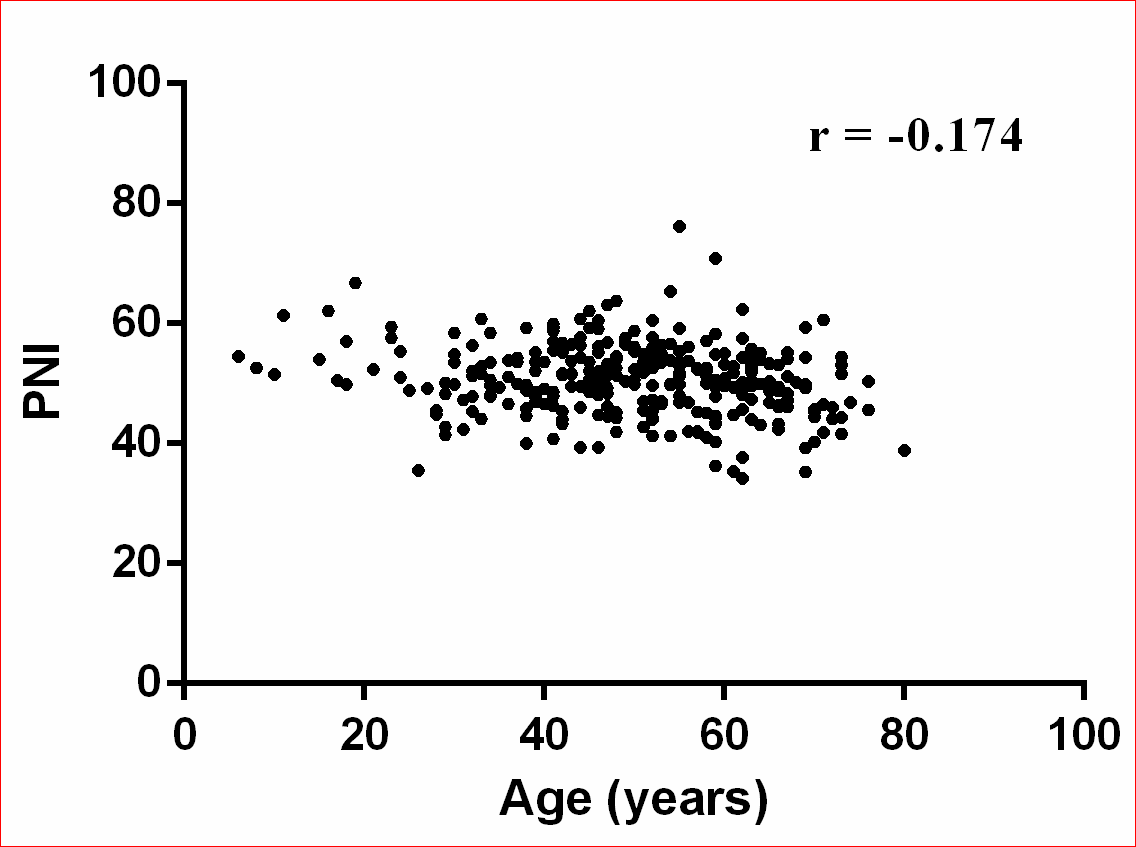

Supplement: Supplemental Digital Content [file medi-97-e13218-s001.doc]
